# The transport of mannitol in *Sinorhizobium meliloti* is carried out by a broad-substrate polyol transporter SmoEFGK and is affected by the ability to transport and metabolize fructose

**DOI:** 10.1099/mic.0.001371

**Published:** 2023-07-28

**Authors:** MacLean G. Kohlmeier, Ivan J. Oresnik

**Affiliations:** ^1^​ Department of Microbiology, University of Manitoba, Winnipeg, MB, Canada

**Keywords:** Rhizobium, metabolism, polyol, ABC transporter

## Abstract

The *smo* locus (sorbitol mannitol oxidation) is found on the chromosome of *S. meliloti *’s tripartite genome. Mutations at the *smo* locus reduce or abolish the ability of the bacterium to grow on several carbon sources, including sorbitol, mannitol, galactitol, d-arabitol and maltitol. The contribution of the *smo* locus to the metabolism of these compounds has not been previously investigated. Genetic complementation of mutant strains revealed that *smoS* is responsible for growth on sorbitol and galactitol, while *mtlK* restores growth on mannitol and d-arabitol. Dehydrogenase assays demonstrate that SmoS and MtlK are NAD^+^-dependent dehydrogenases catalysing the oxidation of their specific substrates. Transport experiments using a radiolabeled substrate indicate that sorbitol, mannitol and d-arabitol are primarily transported into the cell by the ABC transporter encoded by *smoEFGK*. Additionally, it was found that a mutation in either *frcK*, which is found in an operon that encodes the fructose ABC transporter, or a mutation in *frk*, which encodes fructose kinase, leads to the induction of mannitol transport.

## Introduction

*Sinorhizobium meliloti* is a Gram-negative α-proteobacterium that can establish a symbiotic nitrogen-fixing relationship with legumes of the genus *Medicago* [1]. The bacteria’s ability to utilize different sources of carbon for growth has been shown to influence its symbiotic performance, making bacterial carbon metabolism a point of interest for studies on symbiosis [2–5].

Sugar alcohols have been determined to be a preferred carbon source for rhizobia [6, 7]. The catabolism of sugar alcohols such as inositol, glycerol and erythritol have been shown to effect competition for symbiosis [5, 8–10]. However, despite sorbitol, mannitol and galactitol being some of the most commonly encountered sugar alcohols in vascular plants [11], and the use of mannitol as a media component for the isolation of rhizobia from soil [12], the genetic determinants of sorbitol and mannitol metabolism in *S. meliloti* are poorly understood.

Biochemical enzyme activities for sorbitol dehydrogenase, mannitol dehydrogenase and d-arabitol dehydrogenase have been demonstrated from protein extracts of *S. meliloti* grown on sugar alcohols [13, 14]. Fructose kinase activity has also been observed and mutant strains lacking this activity are unable to grow using sorbitol, mannitol or fructose as a sole carbon source suggesting that metabolism of these polyols proceeds using fructose as an intermediate [15]. Similarly, phosphoglucose isomerase (*pgi*) mutants are unable to grow using sorbitol, mannitol or fructose as sole carbon sources [16]. These carbon phenotypes and enzymatic activities suggest that sorbitol and mannitol are oxidized into fructose, which is phosphorylated into fructose-6-phosphate (F6P), and subsequently isomerized into glucose-6-phosphate (G6P) in *S. meliloti* [15].

A set of ATP-binding cassette (ABC) transport genes responsible for fructose uptake have been localized in *S. meliloti* [17]. As well, a transporter capable of transporting the polyols erythritol adonitol, and l-arabitol has also been previously characterized [18]. ABC transporters are the most common transporters in the *S. meliloti* genome [19]. They are generally composed of a solute-binding protein (SBP), a transmembrane domain (TMD) that spans the membrane bilayer and a nucleotide-binding domain (NBD) found in the cytoplasm. SBPs are the major determinate of substrate specificity for ABC uptake systems and exhibit high-affinity substrate binding, with dissociation constants often in the sub-micromolar range [20].

Although the annotation of the *S. meliloti* genome contains a locus annotated as *smo* (**s** orbitol **m** annit **o** l utilization) [21], the genetic components that contribute to sorbitol and mannitol catabolism have not been precisely identified or investigated. The purpose of this study is to characterize the genetic determinates of sorbitol and mannitol transport and metabolism in *S. meliloti *.

## Methods

### Bacterial strains, plasmids and culture conditions

Strains and plasmids used in this study are listed in Table S1 (available in the online version of this article). *meliloti* strains were grown at 28 °C on either Luria–Bertani (LB) [22] or LB^MC^ (LB amended with 2.5 mM MgSO_4_ and 2.5 mM CaCl_2_ [3]) medium as a complex medium or Vincent’s minimal medium (VMM) as a defined medium [12]. Filter sterilized carbon sources were added to the sterile medium to a final concentration of 15 mM. Growth of strains on agar plates was scored semi-quantitatively relative to the wild-type. In the case of ambiguity, experiments were repeated and/or compared to growth in broth cultures using optical density. *Escherichia coli* strains were grown at 37 °C on LB medium. Filter sterilized antibiotics were added to the medium as required at the following concentrations: streptomycin (Sm) 200 μg ml^−1^, neomycin (Nm) 200 μg ml^−1^, gentamicin (Gm) 20 or 60 μg ml^−1^, kanamycin (Km) 20 μg ml^−1^, chloramphenicol (Cm) 20 μg ml^−1^, tetracycline (Tc) 5 μg ml^−1^. Strains that were constructed were single colony purified three times prior to use.

### DNA manipulations and genetic techniques

Standard techniques were used for plasmid isolation, restriction enzyme digests, ligations, transformations and agarose gel electrophoresis [23]. Conjugations and transductions were carried out essentially as previously described [3, 24]. Tn*5* mutagenesis was performed as previously described [25]. The point of insertion was determined by using an arbitrary PCR protocol [26].

The cosmid pJD06 was isolated by complementation of SMc01500 for growth on sorbitol using the cosmid bank CX1 as previously described [27, 28]. Strains SRmD491, SRmD492, SRmD493 and SRmD495 were generated by mutagenesis of pJD06 with Tn*5-*B20 using the strain EcA101 [29]. Briefly, pJD06 was conjugated into EcA101, which carries a chromosomally localized Tn*5*-B20, and subsequently reintroduced into SMc01500 and screening the transconjugants for lack of complementation on sorbitol. Plasmids pMK8, 12, 14 and 17 were found to carry inserts in *smoE*, *smoF*, *smoK* and *smoS,* respectively. These plasmids were then introduced into Rm1021, and allelic exchange was carried out using pPH1JI [26].

Strains SRmD616, SRmD641 and SRmD654 were generated by targeted mutagenesis using either pKNOCK-Gm [30] or pKan [31]. An internal fragment from the gene of interest was PCR amplified using primers *frk*_pK_F/R, *smoC*_pK_F/R, and *pgi*_F1/R1 and cloned into pKNOCK-Gm or pKan (Table S1). Constructs were conjugated into Rm1021, and recombinants were selected using the appropriate antibiotic selection. Recombinants were purified and verified prior to use.

Plasmids pJD02, pMK38, and pMK39 were generated using the ORFeome Gateway system [32, 33] using pCO37 as a destination vector [18]. Genes expressed from pCO37 were constitutively expressed from a P_lac_ promoter. To generate pMK48, *frk* was PCR amplified as a *Hind*III/*Eco*RI fragment using primers *frk*_F2/*frk*_R2, which contain a ribosome-binding site and 6xHis tag to facilitate expression and purification, respectively (Table S1). This fragment was cloned into pRK7813 such that it was constitutively expressed in *S. meliloti* by the P_lac_ promoter [34].

### Protein purification and biochemical enzyme assays

Rm1021 cells expressing pMK48 were grown in 1 l of LB, shaking, at 30 °C for 2 days. The cells were then harvested by centrifugation (6000 ***g*** for 10 min), resuspended in buffer (50 mM TRIS pH 8.0, 300 mM NaCl, 2 mM DTT, 10 mM imidazole), and lysed using a French Press (16 000 lb/in^2^). The lysate was cleared by centrifugation (6000 ***g*** for 10 min). The cleared lysate was then loaded onto Ni-NTA column, washed with ten column volumes of lysis buffer, and eluted from the column with 500 mM imidazole. Eluted fractions were separated by SDS-PAGE and visualized by staining with Coomassie Brilliant Blue. Fractions containing the protein of interest were dialysed overnight in 2 l dialysis buffer (20 mM HEPES pH 7.5, 150 mM NaCl, 2 mM DTT) prior to being used to determine kinase activity. Fructose kinase assays were conducted essentially as previously described [35]. Fructose kinase activity was coupled to the production of NAD^+^ by pyruvate kinase (PK) and lactate dehydrogenase (LDH), which was measured at 340 nm for 2 min in a buffer containing 60 mM HEPES pH 7.5, 6 mM MgCl_2_, 3 mM ATP, 3 mM PEP, 0.3 mM NADH and 1/50 vol PK/LDH mix (Sigma). The assay was initiated with 6 mM substrate, either fructose or glucose. Measured rates of NADH oxidation were linear over a 2 min period and proportional to the volume of extract that was used in the assay.

In-gel dehydrogenase assays were conducted as previously described [36]. Briefly, cell-free extracts were separated by nondenaturing polyacrylamide gel electrophoresis and the gels were stained for dehydrogenase activity using an assay reagent containing phenazine methosulfate, nitro blue tetrazolium salts, NAD^+^ and a substrate of interest.

### Transport assays

Radioactive ^14^C-mannitol and ^14^C-fructose were purchased from American Radiolabeled Chemicals (St. Louis, Mo). Transport rates were determined as previously described [28, 37, 38]. Cultures were grown in defined medium containing a carbon of interest to an OD_600_ between 0.6 and 0.8. The cultures were then washed twice and resuspended to an OD_600_ of 0.3 in defined salts medium. Assays were initiated by the addition of ^14^C-mannitol or ^14^C-fructose to a final concentration of 2 µM. Competing substrates were added to a final concentration of 2 or 10 µM. Subsequently, culture aliquots were passed through a 0.45 µm Hv filter on a Millipore sampling manifold at specified time points. Accumulation of radiolabel was quantified using a liquid scintillation counter (Beckman LS6500). Uptake rates were standardized to total protein.

### Fluorescence gene-expression analysis

Strains were grown overnight in 5 ml cultures at 30 °C using LB^MC^ medium with appropriate antibiotics. These cells were subcultured into 3 ml LB containing 200 µg µl^–1^ Gm medium, induced with 15 mM sorbitol, and grown for 2 days. A volume of 100 µl of these cultures was aliquoted into 96-well black Greiner microplates. Cells were quantitated at OD_600_, fluorescence was measured (485 nm excitation/510 nm emission) using a SpectraMax M2 microplate reader (Molecular Devices, Sunnyvale, CA, USA). Relative fluorescence was determined as (fluorescence at 485/510 – background)/OD_600_. Values obtained from Rm1021 were used as background [39].

## Results

### *smoS* is responsible for growth with sorbitol and galactitol while *mtlK* is responsible for mannitol and d-arabitol utilization

A putative polyol dehydrogenase gene, *smoS*, has been identified by a genome wide screen of short-chain dehydrogenase genes in *S. meliloti* [40]. It has been shown that polar mutations in *smoS* result in reduced growth on sorbitol, mannitol and maltitol [40] as well as galactitol [36]. The gene encoding SmoS is found within a group of genes that has been annotated as being involved in sorbitol and mannitol catabolism based on its similarity to *Rhodobacter sphaeroides* [41].

**Fig. 1.**
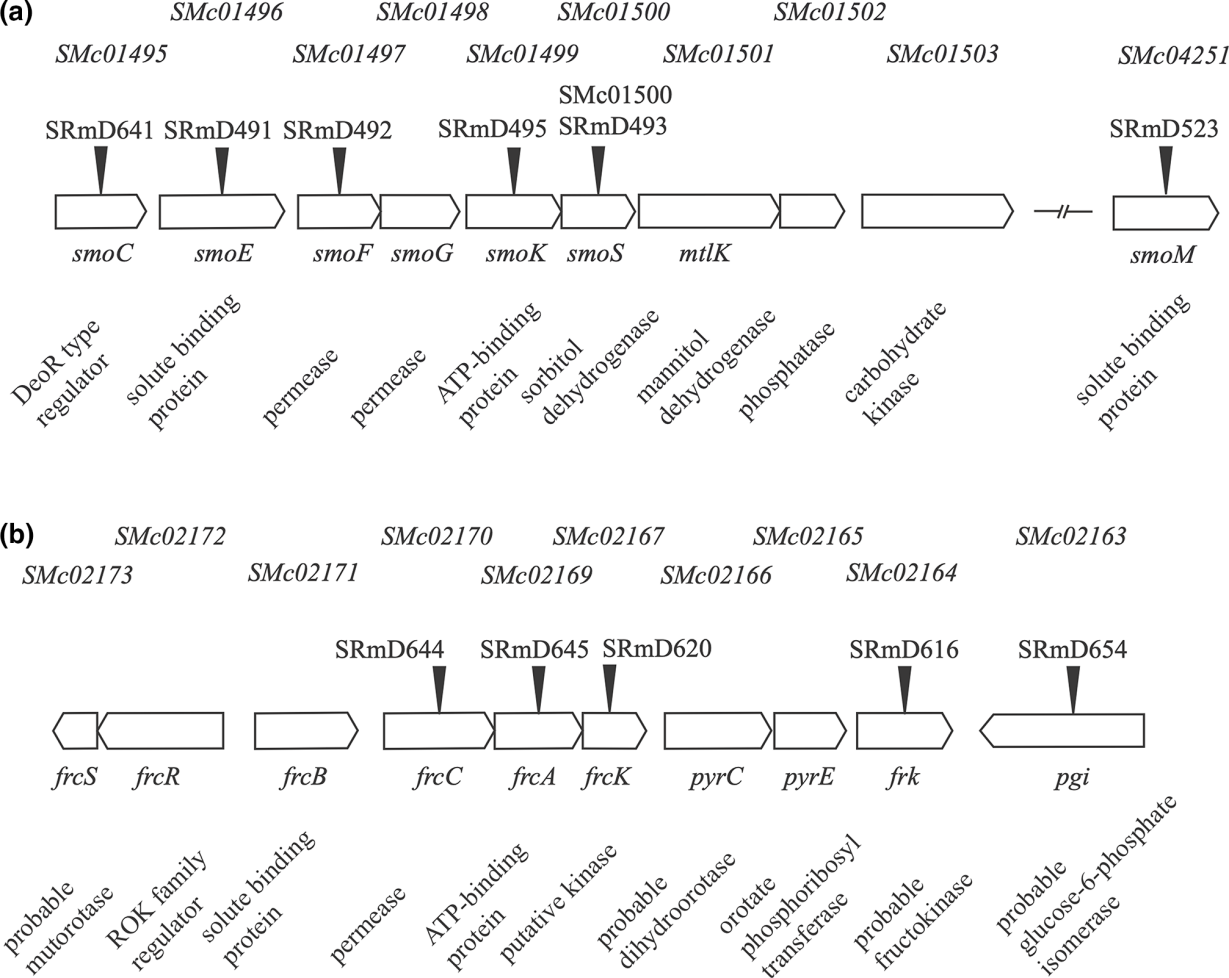
Locus diagrams of the *smo* (**a**) and *frc* (**b**) loci. Locus tags appear above the genes and annotation information appears below. Black wedges represent the sites of insertional mutagenesis. Strain designations are listed above these indicators. Note that *SMc04251,* which is not at the *smo* locus within the Rm1021 genome, is included in (**a**) because it was annotated as *smoM* implying that it was related to sorbitol/mannitol utilization.

In *S. meliloti* the region is found on the chromosome and consists of between 7–9 putative genes including a DeoR-type regulator (encoded by *smoC*), an ABC transporter consisting of a solute binding protein (encoded by *smoE*), two permeases (encoded by *smoF* and *smoG*), an ATP binding protein (encoded by *smoK*), as well as a sorbitol dehydrogenase (encoded by *smoS*) and a mannitol dehydrogenase (encoded by *mtlK*). Two other genes that may be part of this region are *SMc01502* (encoding a putative phosphatase hydrolase) and *SMc01503* (encoding a putative carbohydrate kinase) (Fig. 1a). These latter two genes are absent from the region in *R. sphaeroides *.

We have previously shown that SmoS is capable of galactitol oxidation [36], but its role in sorbitol and mannitol metabolism was unknown. To better characterize this region and its role in polyol catabolism in *S. meliloti *, a cosmid capable of complementing the *smoS* mutant strain for growth on sorbitol was isolated. The cosmid, pJD06, was mutagenized using Tn*5*-B20 and the insertions were subsequently recombined into the chromosome (Table 1). The results of the mutagenesis ultimately yielded strains SRmD491, SRmD492, SRmD493 and SRmD495, which were found to have insertions within *smoE*, *smoF*, *smoS* and *smoK,* respectively (Fig. 1a).

**Table 1.** Bacterial strains and plasmids

Strain or plasmid	Relevant characteristics	Reference
Strains		
* S. meliloti *		
Rm1021	SU47 *str-21*, Sm^r^	[53]
RmP110	Rm1021 *pstC^+^*, Sm^r^	[54]
SMc01500	RmP110 *smoS*::pTH1703, Gm^r^	[40]
FL4643	RmP110 *mtlK*::pTH1522, Gm^r^	[39]
SRmA723	Rm1021 *SMc01627*::Tn*5*-233, Gm^r^Sp^r^	[45]
SRmD491	Rm1021 *smoE*::Tn*5*-B20, Nm^r^	[36]
SRmD492	Rm1021 *smoF*::Tn*5*-B20, Nm^r^	[36]
SRmD493	Rm1021 *smoS*::Tn*5*-B20, Nm^r^	[36]
SRmD495	Rm1021 *smoK*::Tn*5*-B20, Nm^r^	[36]
SRmD501	ΦSRmD491→SRmA723	This study
SRmD502	ΦSRmD492→SRmA723	This study
SRmD503	ΦSRmD495→SRmA723	This study
SRmD523	Rm1021 *smoM*::pKNOCK-Gm, Gm^r^	[36]
SRmD524	ΦSRmD491→SRmD523	This study
SRmD525	ΦSRmD492→SRmD523	This study
SRmD526	ΦSRmD495→SRmD523	This study
SRmD616	Rm1021 *frk*::pKNOCK-Gm, Gm^r^	This study
SRmD618	Rm1021 *frcK*::pKan, Nm^r^	This study
SRmD620	Rm1021 *frcK*::Tn*5*, Nm^r^	This study
SRmD641	Rm1021 *smoC*::pKan, Nm^r^	This study
SRmD642	ΦSRmD641→FL4643	This study
SRmD654	Rm1021 *pgi*::pKNOCK-Gm, Gm^r^	This study
SRmD664	Rm1021 *frcA*::pKNOCK-Gm, Gm^r^	This study
SRmD665	Rm1021 *frcC*::pKNOCK-Gm, Gm^r^	This study
SRmD666	ΦSRmD495→SRmD664	This study
SRmD667	ΦSRmD495→SRmD665	This study
* E. coli *		
DH5α	F^-^ *supE44 lacU169 hsdR17 recA1 endA1 gyrA96 thi-1 relA1* (80*lacZ*ΔM15)	[55]
DH5αRλpir	λpir lysogen of DH5α	[56]
MM294A	*pro-82-thi-1 hsdR17 supE44*	[57]
MT607	MM294A *recA56*	[57]
MT616	MT607 (pRK600)	[57]
EcA101	MT607ΩTn*5*-B20, Km^R^	[58]
Plasmids		
pSMc01500	*smoS*/pCO37, Tc^r^	[36]
pJD02	*mtlK*/pCO37, Tc^r^	This work
pJD06	CX1 derived sorbitol/mannitol complementing cosmid, Tc^r^	This work
pMK8	pJD06 *smoE*::Tn*5*-B20	This work
pMK12	pJD06 *smoF*::Tn*5*-B20	This work
pMK13	pJD06 *smoS*::Tn*5*-B20	This work
pMK18	pJD06 *smoK*::Tn*5*-B20	This work
pMK38	*frk*/pCO37, Tc^r^	This work
pMK39	*frcK*/pCO37, Tc^r^	This work
pMK48	6xHis*frk*/pRK7813, Tc^r^	This work
pMK62	*pgi*/pCO37, Tc^r^	This work
pRK7813	Broad host range vector, Tc^r^	[59]
pCO37	Gateway compatible vector, Tc^r^	[40]
pRK600	pRK2013 *nptI*::Tn*9*, Cm^r^	[57]
pRK602	pRK600ΩTn*5*, Cm^r^ Nm^r^	[25]
pPH1JI	IncP plasmid, Gm^r^	[60]
pKan	Suicide vector, Km^r^	[31]
pKNOCK-Gm	Suicide vector, Gm^r^	[30]
pTH1522	Reporter vector, *gfp^+^*, *lacZ*, *gusA*, *tdimer2(12*), Gm^r^	[39]

a Sm^r^, streptomycin resistant; Nm^r^, neomycin resistant; Gm^r^, gentamicin resistant; Sp^r^, spectinomycin resistant; Cm^r^, chloramphenicol resistant; Tc^r^, tetracycline resistant; ΦM12 transducing lysates are indicated by Φ preceding the strain number. For strain constructions, an arrow indicates transduction from the lysate into the recipient strain.

The gene *smoE*, encoding a SBP, has been shown to be induced by sorbitol, mannitol and maltitol [42]. When SRmD491, SRmD492, SRmD493 and SRmD495 were streaked on defined medium it was found that growth on sorbitol and mannitol is nearly abolished (Table 2). d-arabitol was also tested because of its structural similarity to mannitol, and the mutant strains were similarly unable to utilize d-arabitol for growth (Table 2). Introduction of pSMc01500, which carries a wild-type copy of *smoS,* into either SMc01500 or SRmD493, which have insertions in *smoS*, resulted in the restoration of growth on sorbitol but not mannitol or d-arabitol. The introduction of pJD02, which carries a wild-type copy of *mtlK*, resulted in the restoration of growth on mannitol and d-arabitol (Table 2). Introduction of pSMc01500 into SRmD491, SRmD492, or SRmD495 which carry mutations in the associated ABC transporter resulted in the restoration of growth on defined medium with sorbitol. Similarly, the introduction of pJD02 into SRmD491, SRmD492 and SRmD495 restored the ability to grow on mannitol and d-arabitol. This suggests that the genes from *smoE* to *mtlK* are part of a monocistronic operon whereby insertional disruption of *smoEFGK* has polar effects on these downstream metabolic genes (Table 2). It also suggests that the SmoEFGK transporter is not necessary for growth on sorbitol, mannitol and d-arabitol. Either SmoEFGK does not transport these substrates, or there must exist an alternate transporter in the genome that is capable of recognizing sorbitol, mannitol, as well as d-arabitol and providing physiologically relevant transport if the downstream catabolic gene is provided on a multicopy plasmid (Table 2).

**Table 2.** Complementation analysis of mutations in the *smo* locus

Strain	Relevant genotype Chromosomal (plasmid)	LB	sbt	mtl	d-atl	fru	gly
Rm1021	Wild-type	+	+	+	+	+	+
SRmD491	*smoE*	+	+/−	+/−	+/−	+	+
SRmD491 (pSMc01500)	*smoE* (*smoS*)	+	+	+/−	+/−	+	+
SRmD491 (pJD02)	*smoE* (*mtlK*)	+	+/−	+	+	+	+
SRmD492	*smoF*	+	+/−	+/−	+	+	+
SRmD492 (pSMc01500)	*smoF* (*smoS*)	+	+	+/−	+	+	+
SRmD492 (pJD02)	*smoF* (*mtlK*)	+	+/−	+	+	+	+
SRmD493	*smoS*	+	−	−	+/−	+	+
SRmD493 (pSMc01500)	*smoS* (*smoS*)	+	+	−	+/−	+	+
SRmD493 (pJD02)	*smoS* (*mtlK*)	+	−	+	+	+	+
SRmD495	*smoK*	+	+/−	+/−	+/−	+	+
SRmD495 (pSMc01500)	*smoK* (*smoS*)	+	+	+/−	+/−	+	+
SRmD495 (pJD02)	*smoK* (*mtlK*)	+	+/−	+	+	+	+

Strains were streaked onto complex (LB) or defined (VMM) agar medium containing caron sources as indicated in the table. Growth was scored as follows; +, like wild-type; -, no growth; +/−, poor growth. Abbreviations are as follows; LB, Luria–Bertani; sbt, sorbitol; mtl, mannitol; d-atl, d-arabitol; fru, fructose; gly, glycerol.

### SmoS and MtlK are polyol dehydrogenases

The catabolism of polyols is most commonly initiated by the oxidation at the C2 position to yield a keto-sugar that is subsequently phosphorylated [36, 43]. Sorbitol and mannitol are epimers, and oxidation of either of these substrates at the C2 position yields fructose. Although d-arabitol is a pentitol rather than a hexitol, it shares the same stereochemistry with mannitol over its terminal three carbons, suggesting that it too can be oxidized in a similar manner.

**Fig. 2.**
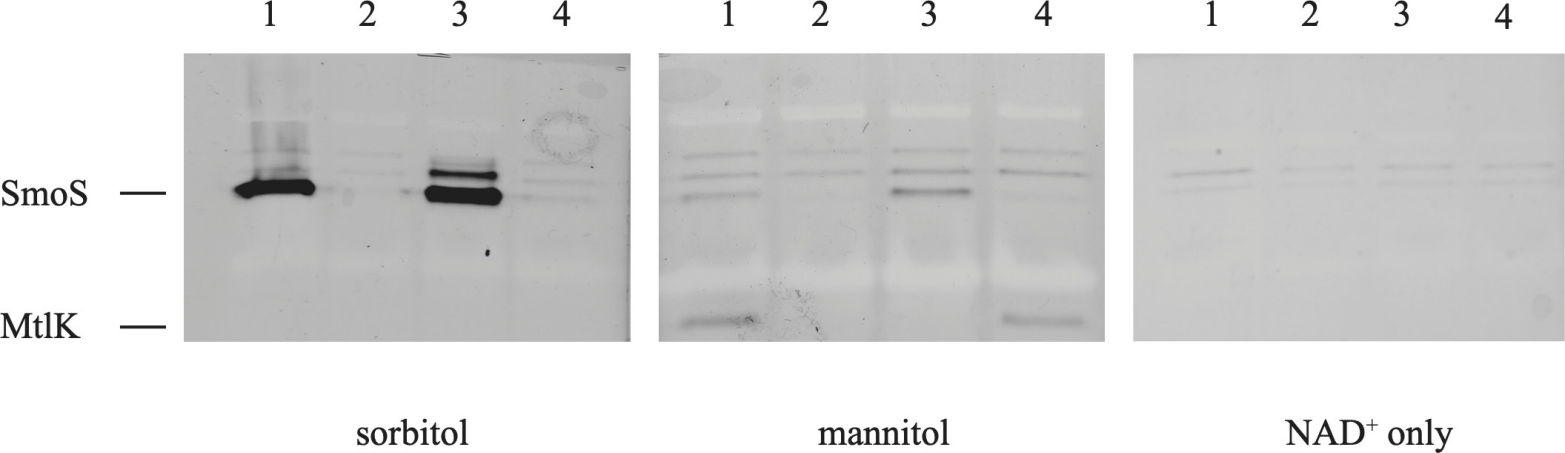
Non-denaturing PAGE gel of sorbitol inducible dehydrogenase activity. Extracts from wild-type (lane 1), SMc01500 (lane 2), SMc01500 with *smoS in trans* (lane 3), and SMc01500 with *mtlK in trans* (lane 4) were grown in glycerol and induced with sorbitol for 6 h. Gels were stained for dehydrogenase activity using an assay reagent containing p-nitroblue tetrazolium, phenazine methosulfate and NAD^+^. The substrate added is listed below each panel.

To provide evidence to corroborate this hypothesis wild-type cells were grown in defined medium with glycerol as a sole carbon source and induced with sorbitol for 6 h. The cultures were lysed, and the protein extracts were then separated using nondenaturing polyacrylamide gel electrophoresis. The gels were assayed for enzyme activities by staining for dehydrogenase activity and compared with a control gel that did not have exogenous carbon added during the assay (Fig. 2).

The data show that when the separated wild-type extract is incubated with sorbitol a distinct band is found, whereas when it is incubated with mannitol a separate faster migrating band is observed (Fig. 2, lane 1). These bands of activity were absent in extracts generated from the *smoS* mutant strain SMc01500 (Fig. 2, lane 2). Introduction of either pSMc01500, which carries a wild-type copy of *smoS*, into the mutant strain, or pJD02, which carries a copy of the wild-type *mtlK*, restored the presence of either a sorbitol (Fig. 2, lane 3) or mannitol (Fig. 2, lane 4) oxidizing protein within the extract. These bands of activity mimic the intensity and migration distance of the wild-type bands. Of note, if d-arabitol was used as a substrate, bands that are coincident with what mannitol result were observed (data not shown). Taken together, these data suggest that *smoS* encodes an NAD^+^-dependent sorbitol and galactitol dehydrogenase while *mtlK* encodes an NAD^+^-dependent mannitol and d-arabitol dehydrogenase. We note that SmoS from *S. meliloti* has been recently crystalized and biochemically characterized [14].

### Sorbitol and D-arabitol strongly compete with mannitol for transport

The polyols erythritol, adonitol and d-arabitol are all transported by a single ABC transporter (multiple polyol transporter) that is encoded by *mptABCDE* in *S. meliloti* [18]. Additionally the transport of mannitol has been shown to be inducible by mannitol, and it was shown that galactitol could directly compete with mannitol transport [36].

**Fig. 3.**
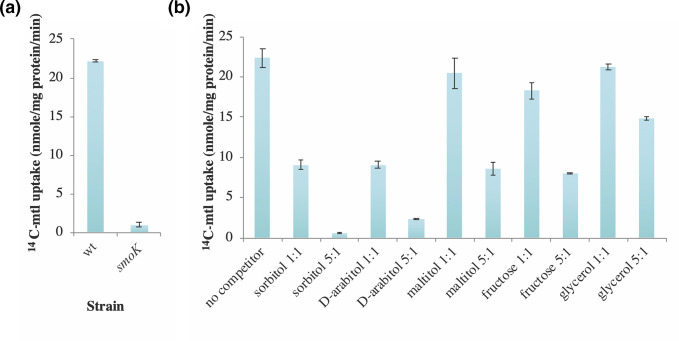
Transport rates of ^14^C-mannitol. (**a**) Rm1021 (wild-type) and SRmD495 (*smoK*::Tn*5*-B20) grown in mannitol and glucose, 2 µM labelled mannitol was used to initiate the assay. (**b**) Uptake of ^14^C-mannitol by Rm1021 in competition with unlabeled substrates. Cells were grown on mannitol as a sole carbon source. 2 µM labelled mannitol was competed against either 2 µM or 10 µM unlabeled substrate. Accumulation of label is shown in nmole/mg protein/min. Data are expressed as the mean±sd of three independent replicates.

To determine the extent to which the SmoEFGK transport system contributes to sugar alcohol uptake, we tested the ability of SRmD495, a strain carrying an insertion mutation in the ATP-binding protein of the ABC transporter to take up ^14^C labelled mannitol. The wild-type, Rm1021, as well as the SRmD495 were grown in defined medium with glucose and mannitol as carbon sources. The strains were subsequently assayed for their ability to accumulate radiolabeled mannitol. The results show that SRmD495 cells were severely compromised in their ability to transport mannitol in comparison to the wild-type (Fig. 3a), suggesting that the SmoEFGK transport system is involved in the uptake of mannitol.

The transport of ^14^C mannitol by Rm1021 has been shown to be inducible and that galactitol could compete for transport using the mannitol transporter [36]. To determine if sorbitol, and d-arabitol were capable of being taken up by the same transporter, these sugar-alcohols were competed against labelled mannitol in transport assays (Fig. 3b). The data show that when sorbitol or d-arabitol were mixed with ^14^C-mannitol in equal amounts, labelled mannitol uptake rates were reduced by approximately 50 % (Fig. 3b). If the concentration of unlabeled sorbitol or d-arabitol is in great excess of the ^14^C-mannitol concentration (5 : 1), uptake rates are nearly abolished (Fig. 3b). This suggests that sorbitol and d-arabitol utilize the same transporter as mannitol which is predominately conducted by the SmoEFGK system.

Maltitol, fructose and glycerol were also tested for their ability to inhibit the uptake of mannitol. Maltitol is a disaccharide made up of sorbitol and glucose monomers. Maltitol has been shown to induce *smoE* [42] and mutations in *smoS* exhibit reduced growth on maltitol as a sole carbon source [40]. In addition, since both mannitol and sorbitol are ultimately converted to fructose, these substrates, as well as glycerol, which has the same stereochemistry were also competed against mannitol. The results show that with either maltitol or fructose at a 1 : 1 ratio with mannitol there was a slight reduction in mannitol transport. Whereas the maltitol data is not statistically significant, the fructose decrease was significant (*P*=0.03). There was no visible reduction in transport of mannitol using glycerol at this ratio (Fig. 3b). When the ratio of these substrates was increased to be 5 : 1, it was found that both maltitol and fructose decreased transport rates of mannitol to less than 50 % of the wild-type rate, whereas the glycerol reduction was approximately 30 % (Fig. 3b).

### The *smo* locus is negatively regulated by SmoC

The *smo* locus contains a putative regulator annotated as *smoC* (*SMc01495*) that seems likely to be involved in the regulation of the operon (Fig. 1a). A comparison of the SmoC amino acid sequence to other proteins encoded by the Rm1021 genome revealed a sequence similarity (E value of 1×10^−56^) to EryD, a DeoR-type regulator. Regulators of the DeoR family typically negatively regulate the genes under their control [44]. However, an *S. meliloti* strain carrying an *eryD* mutation was unable to grow using erythritol as a sole carbon source and, based on qRT-PCR data, was consistent with it acting as both a positive and a negative regulator of erythritol catabolism [45].

To determine how SmoC effects the transcription of the *smo* operon, a *smoC* mutant, SRmD641, was constructed. The strain FL4643 was identified in a *S. meliloti* fusion library as having a *gfp^+^* gene transcriptionally fused to the 3′ end of *mtlK* [ +]. To determine if SmoC could regulate this operon the mutant *smoC* allele was transduced into a FL4643 background, generating strain SRmD642.

**Fig. 4.**
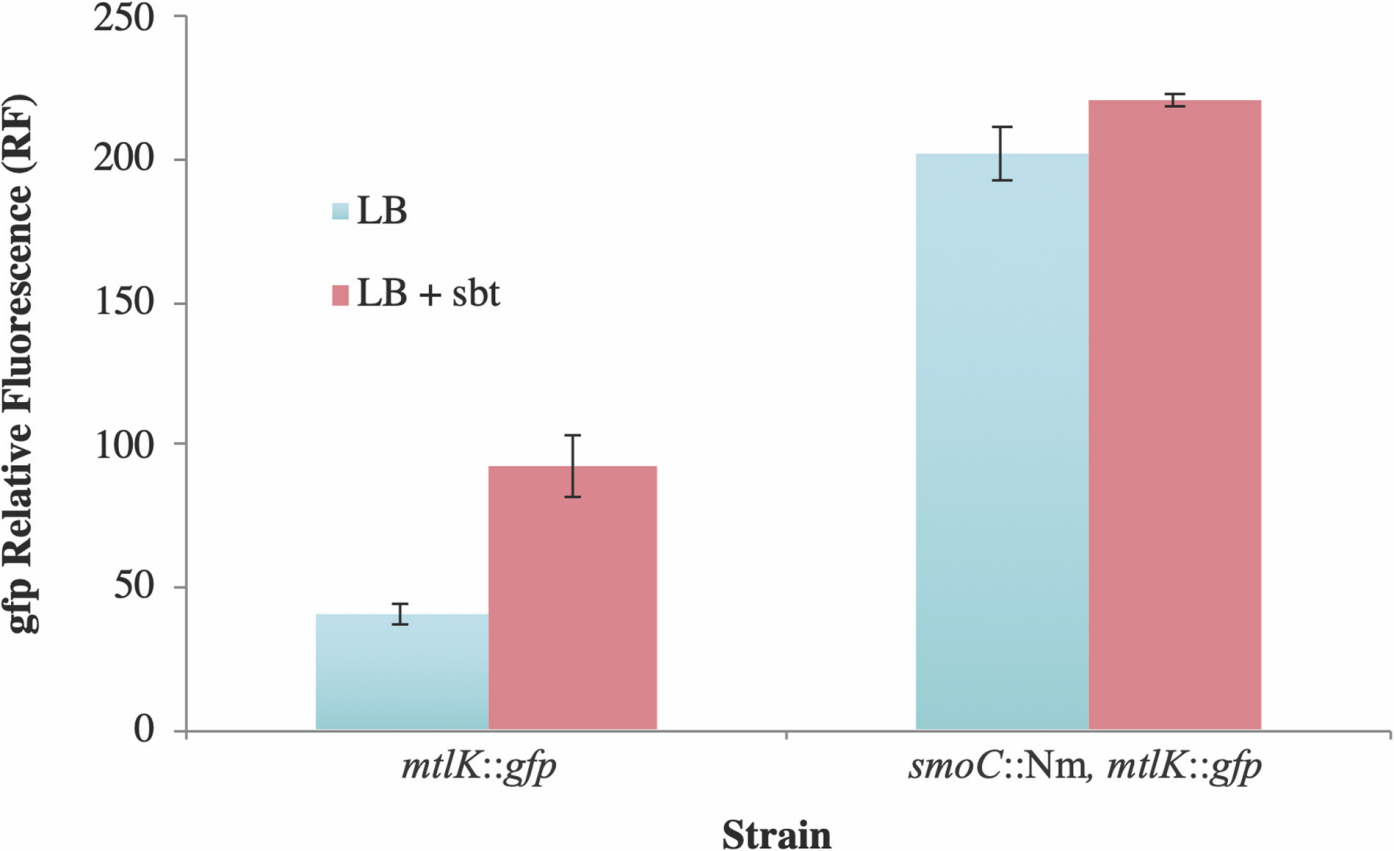
Induction of the *smo* locus measured by GFP relative fluorescence (RF). Strains were grown in LB broth either with, or without sorbitol (sbt) as described in Methods. GFP was read at wavelengths of 485 (excitation) and 510 (emission). RF=(fluorescence-background)/OD600. Strains used are *mtlK::gfp*, FL4643; *smoC*::Nm, *mtlK::gfp*, SRmD642. Rm1021 was used to control for background fluorescence. The data is shown as the average ±sd of three biological replications.

The results show that expression of *gfp* increased when FL4643 cells were grown in the presence of *smo* inducer substrates such as sorbitol (Fig. 4), consistent with previous results suggesting that transport was inducible by mannitol [36]. *gfp^+^* + of SRmD642 was greater than double that observed for sorbitol induced FL4643 regardless of the growth conditions (Fig. 4). Collectively, the data suggest that SmoC functions as a negative regulator, and that mutations to *smoC* result in constitutive expression of the *smo* operon. Additionally, the data also show that *smoC* is independently transcribed since the disruption of *smoC* did not have polar effects on *mtlK* (Fig. 1a).

### *Frk* encodes a fructose kinase

The oxidation of sorbitol and mannitol by SmoS and MtlK, respectively, generate fructose. To enter central carbon metabolism fructose needs to be phosphorylated to become F6P, which is then subsequently converted to G6P in *S. meliloti* by phosphoglucose isomerase [7, 15, 16]. Whereas enzyme activities and mutants in fructokinase and phosphoglucose isomerase have been previously reported in *S. meliloti* strain L5-30, these were mutants that were generated by chemical mutagenesis prior to the ability to acquire whole-genome sequences [15, 16]. To provide clarity to the genome annotation of *S. meliloti* Rm1021, as well as for characterizing a complete catabolic pathway for sorbitol and mannitol, it was decided to unambiguously ascribe the encoded function to the proper gene(s).

Within the genome annotation there is ambiguity regarding the gene responsible for encoding the fructose kinase in *S. meliloti *. A region, called the *frc* locus (Fig. 1b), has been previously characterized and determined to encode a transporter responsible for the uptake of fructose [17]. A gene encoding a putative kinase, *frcK*, was identified immediately adjacent and presumably in the same operon as *frcCA* since its open reading frame overlaps *frcA* by three base pairs. It is annotated as a kinase but was determined to be an unlikely candidate as a fructose kinase based on low sequence similarity to a characterized fructokinase in *R. leguminosarum* [7, 17, 46]. A second putative kinase, *frk*, is located within 2 kb of the transport genes and *frcK* (Fig. 1b).

To resolve the ambiguity, a screen for mutants unable to utilize fructose was carried out. Rm1021 was mutagenized with Tn*5*, and approximately 2000 colonies were screened for an inability to grow using fructose as a sole carbon source. A single insertion mutant was identified and determined to be within *frcK*. The strain carrying this mutation was designated SRmD620 (Fig. 1b). The *frk* mutation was constructed using pKNOCK-Gm as described, and the strain carrying this mutation was designated SRmD616. These strains were screened for growth on various carbon sources and the results show that a mutation in *frcK* reduces growth on sorbitol, mannitol and fructose, but not on either glycerol or glucose (Table 3). However, mutations in *frk* exhibit a more severe growth deficiency, in which growth is completely abolished, similar to a strain carrying a s*moS* mutation with respect to sorbitol and mannitol (Table 3). Introduction of pMK38, which carries a wild-type copy of *frk*, into either a strain carrying a mutation in *frk* (SRmD616) or *frcK* (SRmD620) was able to fully restore growth of these mutants on all tested carbon sources to wild-type levels, while introduction of pMK39 (carrying *frcK*) into these mutations did not show a growth improvement over the empty vector control (Table 3).

**Table 3.** Complementation of mutants unable to use fructose

Strain	Relevant genotype Chromosomal (plasmid)	LB*	sbt	mtl	fru	gly
Rm1021	Wild-type	+	+	+	+	+
SMc01500	*smoS*	+	−	−	+	+/−
SRmD616	*frk*	+	−	−	−	+
SRmD616 (pMK38)	*frk* (*frk*)	+	+	+	+	+
SRmD616 (pMK39)	*frk* (*frcK*)	+	−	−	−	+
SRmD620	*frcK*	+	+/−	+/−	+/−	+
SRmD620 (pMK38)	*frcK* (*frk*)	+	+	+	+	+
SRmD620 (pMK39)	*frcK* (*frcK*)	+	+/−	+/−	+/−	+
SRmD654	*pgi*	+	−	−	−	nd†
SRmD654 (pMK62)	*pgi* (*pgi*)	+	+	+	+	nd

Strains were streaked onto complex (LB) or defined (VMM) agar medium containing caron sources as indicated in the table. Growth was generally scored after 4 days growth as follows: +, like wild-type; −, no growth; +/−, intermediate growth.

*Abbreviations: LB, Luria–Bertani; sbt, sorbitol; mtl, mannitol; fru, fructose; gly, glycerol.

†nd, not determined. (Experiments with the *pgi* mutant used glucose rather than glycerol as a neutral carbon source to support growth on defined media.)

The ability to restore growth to strains carrying either *frcK* or *frk* mutations suggests that *frk* encodes a fructokinase and that overexpression of this gene is capable of altering the internal metabolic pool of fructose, thus improving the growth rate of *S. meliloti* (Table 3).

**Fig. 5.**
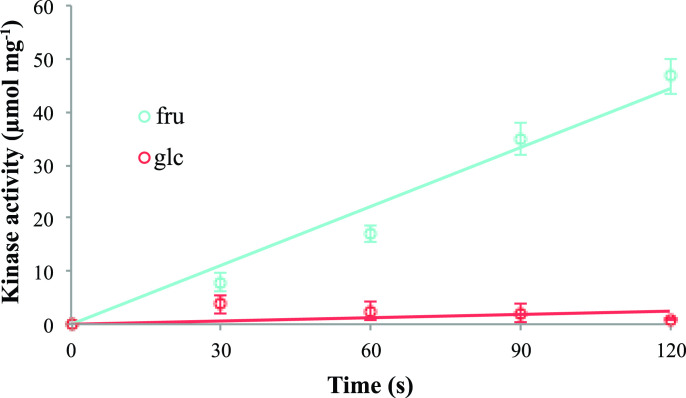
Kinase activity of Frk. Partially purified Frk was assayed for kinase activity using either fructose (fru) or glucose (glu) as substrates. Activity was coupled to the production of NAD^+^ by PK and LDH and was measured at 340 nm for 2 min in a buffer containing 60 mM HEPES pH 7.5, 6 mM MgCl2, 3 mM ATP, 3 mM PEP, 0.3 mM NADH and 1/50 vol PK/LDH mix. Activity is plotted as μmol NAD^+^ produced per milligram protein over time. Data points represent the average ±sd of three biological replications.

To provide evidence for the role of Frk we wished to demonstrate the loss of fructokinase activity in SRmD616. The ability to measure carbohydrate kinase activities in crude extracts can be confounded by high rates of NADH oxidation [36]. This was the case with fructose kinase as initial attempts to measure activity in a crude lysate were ambiguous. As an alternative approach, Frk was N-terminally 6xHis tagged, and expressed from pRK7813 in *S. meliloti* as previously described [47]. The cells were then harvested, and the protein was partially purified using Ni-affinity column. It was found that when the partially purified Frk was assayed for kinase activity using fructose as a substrate it yielded a rate of 17.1 µmol/min/mg protein (Fig. 5). In contrast if glucose was as a substrate to initiate the assay a rate of 2.4 µmol/min/mg were measured, which was equivalent to the background rate of NADH oxidation (Fig. 5).

### Phosphoglucose isomerase gene is downstream of the *frc* locus

There are three genes annotated as encoding a G6P isomerase in the *S. meliloti* genome; *SMc02042* (*pgiA1*), *SMc02163* (*pgi*) and *SMb20857* (*pgiA2*). Of the three, the most likely candidate of these has been assumed to be *pgi* (*SMc02163*), which is found within 10 kb of the *frc* locus. This is based on bioinformatic analysis [7], as well as the results of genome wide Tn-seq guided *in silico* metabolic reconstruction [48]. To provide experimental evidence to support this hypothesis, a strain, SRmD654, carrying an insertional mutation in *pgi* was constructed. A mutation in *pgi* results in a strain that is consistent with previously reported carbon phenotypes [16], chiefly it is unable to grow using sorbitol, mannitol and fructose as a sole carbon source (Table 3). Assaying for phosphogluco-isomerase activity from crude extracts showed that whereas Rm1021 had rates of approximately 9.28 nmol/min/mg, whereas SRmD654 did not have activity over background. Finally, the introduction of pMK62, which carries the wild-type *pgi* resulted in complementation of this mutant (Table 3). Together, these data are consistent with the hypothesis that *SMc02163* encodes the phosphogluco-isomerase used in the upper half of the Embden Meyerhoff Pathway.

### Sorbitol and Mannitol can be transported by the *frc* transporter

That complementation of *smo* transport mutants with *smoS* or *mtlK* can restore growth on sorbitol and mannitol suggests that there is another transporter capable of taking up these substrates (Table 2). Several transport systems were investigated to identify an alternative sorbitol-mannitol transporter. Within the *S. meliloti* genome *SMc04251* is annotated as *smoM*, the mannitol-binding component of a tripartite ATP-independent periplasmic (TRAP) transporter. SRmD523, a *smoM* mutant, was previously constructed to determine if it had a role in galactitol transport [36]. To determine if this gene could play a role in the transport of sorbitol-mannitol the Tn*5*-B20 insertions in *smoE*, *smoF* and *smoK* were transduced into SRmD523 yielding SRmD524, SRmD525 and SRmD526, respectively. It was found that SRmD523 did not have any growth defects on the polyols, and that the additional mutations to the ABC transporter at the *smo* locus did not alter the phenotypes already associated with these genes (data not shown).

We also hypothesized that the range of substrates take up from the multiple polyol transporter (*mpt*) may extend beyond erythritol, l-arabitol and adonitol [38], and may contribute to the uptake of sorbitol, mannitol and d-arabitol. The mutated *smoE*, *smoF* and *smoK* alleles were transduced into a strain containing a mutated ABC-type transporter permease component (*mptB*) generating SRmD501, SRmD502 and SRmD503. These strains exhibited phenotypes identical to the *smo* mutant parental strain with the exception that they could not utilize erythritol for growth (data not shown). Taken together these data suggest that SmoM and MptABCDE do not appear to play a role in sorbitol/mannitol transport.

Growth using mannitol was shown to induce the fructose transport gene *frcC* [17]. Additionally, mannitol transport is reduced in the presence of excess fructose (Fig. 3b). Both implicate the *frcBCA* system in sugar alcohol transport. To determine if the fructose transporter plays a role in sorbitol and mannitol uptake mutations were constructed in *frcA* (SRmD644), as well as *frcC* (SRmD645). To construct strains that were lacking both the fructose and the mannitol transporter the *smoK*::Tn*5*-B20 allele from SRmD495 was transduced into strains carrying either *frcA,* or *frcC* -yielding SRmD666 and SRmD667, respectively (Fig. 1b).

Strains carrying mutations in *frcC*, *frcA* or *frcK* showed a decrease in growth on fructose, mannitol and sorbitol (Tables 3 and 4). A strain carrying a mutation in *smoK* was not impaired for growth using fructose as a carbon source but was unable to efficiently utilize sorbitol or mannitol (Table 3). In contrast, strains carrying mutations in both transporters were completely unable to utilize fructose, sorbitol or mannitol (Table 4). The growth phenotypes suggest that both transporters play a role in the transport of sorbitol, mannitol and fructose into the cell. Complementation of the double transport mutants with *smoS* or *mtlK* did not permit growth on sorbitol or mannitol, suggesting that the *frc* transporter is the second transporter capable of sorbitol and mannitol uptake (Table 4).

**Table 4.** Carbon phenotypes of putative fructose transport mutants

Strain	Relevant genotype Chromosomal (plasmid)	LB	Sbt	Mtl	Fru	Glu
Rm1021	wt	+	+	+	+	+
SRmD495	*smoK*::Tn*5*-B20	+	+/−	+/−	+	+
SRmD664	*frcA*::pKNOCK-Gm	+	+/−	+/−	+/−	+
SRmD665	*frcC*::pKNOCK-Gm	+	+/−	+/−	+/−	+
SRmD666	*smoK*, *frcA*	+	−	−	−	+
SRmD666 (SMc01500)	*smoK*, *frcA* (*smoS*)	+	−	−	−	+
SRmD666 (pJD02)	*smoK*, *frcA* (*mtlK*)	+	−	−	−	+
SRmD667	*smoK*, *frcC*	+	−	−	−	+
SRmD667 (SMc01500)	*smoK*, *frcC* (*smoS*)	+	−	−	−	+
SRmD667 (pJD02)	*smoK*, *frcC* (*mtlK*)	+	−	−	−	+

Strains were streaked onto complex (LB) or defined (VMM) agar medium containing caron sources as indicated in the table. Growth scored as follows; +, like wild-type; -, no growth; +/−, intermediate growth. Abbreviations are as follows: LB, Luria–Bertani; sbt, sorbitol; mtl, mannitol; fru, fructose; glu, glucose.

### FrcK contributes to fructose uptake and mutations to *frcK* or *frk* permit transport of mannitol under non-inducing conditions

To directly assess the role of each transporter in taking up either mannitol or fructose a series of uptake assays were carried out. First, we sought to determine how these transporters were regulated. Previous results have shown that transport of mannitol is inducible, cells grown in mannitol exhibited ^14^C-mannitol uptake rates of 25.8 nmoles/mg protein/min compared to 2.8 nmoles/mg protein/min for cells grown in glucose [36]. To test for induction by fructose, cells were grown overnight in defined medium with fructose and assayed for uptake of ^14^C-mannitol. These cells took up labelled mannitol at a rate of 2.0 nmoles/mg protein/min, suggesting that growth on fructose does not induce the transport of mannitol. We subsequently tested for the uptake of ^14^C-fructose by cells grown in fructose, mannitol or glucose, which exhibited uptake rates of 111.9 nmoles/mg protein/min, 95.1 nmoles/mg protein/min and 16.3 nmoles/mg protein/min, respectively, indicating that growth in fructose or mannitol induces the uptake of fructose. Induction of *frcC* by mannitol has been previously reported [17].

**Fig. 6.**
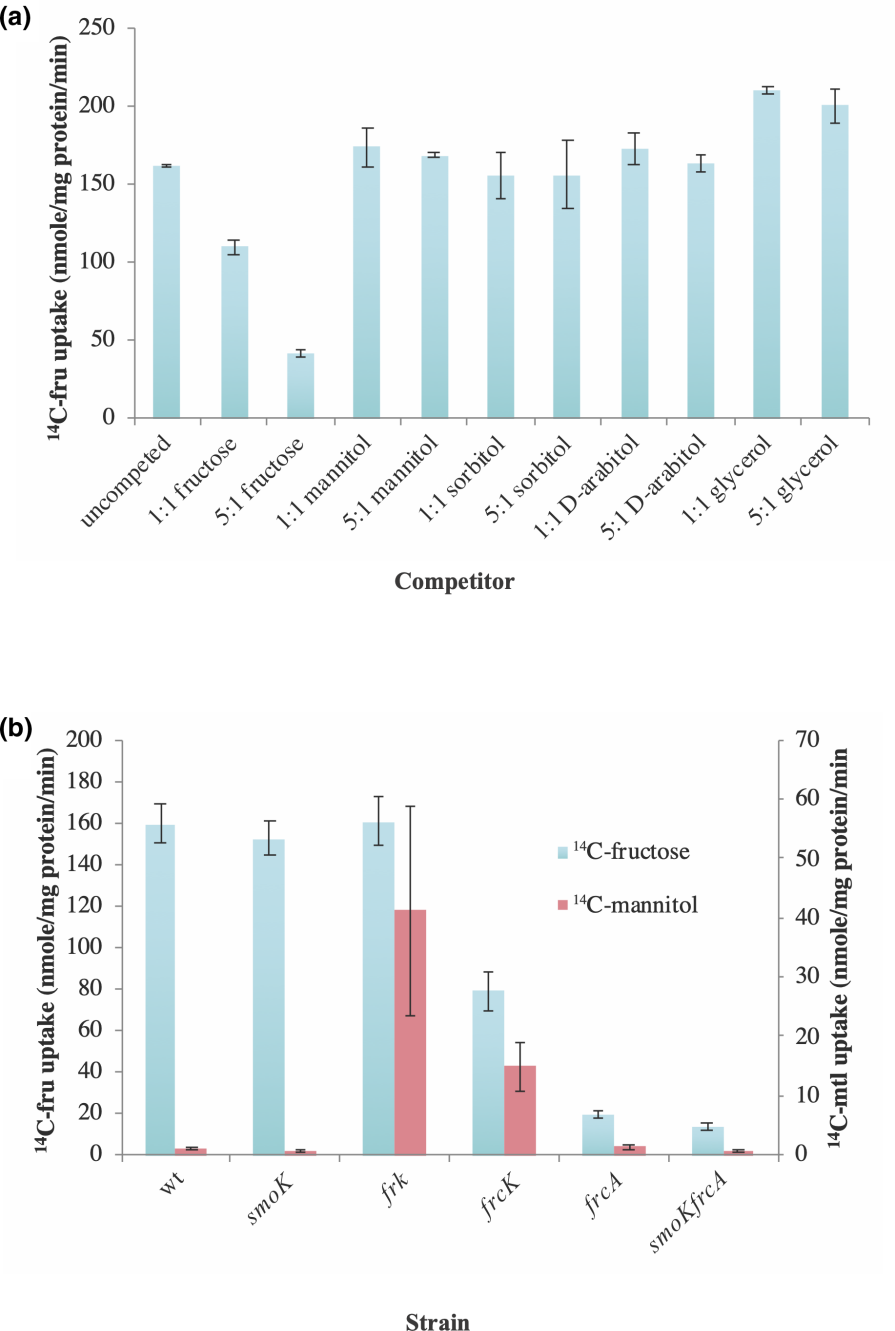
Transport rates of ^14^C-fructose and ^14^C-mannitol. (**a**) Rm1021 in competition with unlabeled substrates. Cells were grown on fructose as a sole carbon source. 2 µM labelled mannitol was competed against either 2 µM or 10 µM unlabeled substrate. (**b**) Uptake of ^14^C-fructose or ^14^C-mannitol by various transport mutants. Cells were grown in fructose and glucose, 2 µM radiolabelled substrate was used to initiate the assay. Accumulation of label is shown in nmole/mg protein/min. Data are expressed as the mean±sd of three independent replicates.

To determine if any of the sugar alcohol substrates could compete with fructose for use of the *frc* transporter, cultures of Rm1021 that were grown in fructose were measured for ^14^C-fructose uptake in the presence of unlabeled fructose, mannitol, sorbitol, d-arabitol and glycerol in equivalent amounts or in fivefold excess. The results show that unlabeled fructose present in a 1 : 1 ratio with the radiolabel can reduce ^14^C-fructose uptake to approximately 65 % of the uncompeted control (Fig. 6a). If unlabeled fructose is present at a ratio of 5 : 1, the uptake of ^14^C-fructose is reduced to 25 % (Fig. 6a). However, none of the sugar alcohol substrates tested were able to reduce the accumulation of ^14^C-fructose regardless of the ratio at which they were present (Fig. 6a). This suggests that sugar alcohols do not utilize the *frc* transporter under the conditions of this assay. It is also possible that sugar alcohols do utilize the *frc* transporter but at too low an affinity to displace fructose from the binding site.

Finally, the transport of ^14^C-fructose and ^14^C-mannitol by different mutant strains was assayed to ascertain the role that these genes play in the transport of fructose and mannitol. Many of the mutants tested grew poorly or not at all using fructose as a sole carbon source, therefore, glucose was added along with fructose to support the growth of these strains. Importantly, transport rates of ^14^C-fructose by the Rm1021 wild-type were high, indicating that growth in the presence of glucose was not inhibiting the uptake of labelled fructose (Fig. 6b). Both the *smoK* and the *frk* mutants can transport fructose at wild-type levels, consistent as the *frc* transport genes are unaffected in these strains (Fig. 6b). The *frcK* mutant exhibited an approximately 50 % reduction in ^14^C-fructose transport rates, suggesting that FrcK does play a role in transport of fructose (Fig. 6b). Additionally, ^14^C-transport rates of the *frcA* and *smoK/frcA* strains are barely above background, indicating that both strains are compromised in their ability to transport fructose (Fig. 6b).

Rm1021 exhibited poor transport rates of ^14^C-mannitol following growth on fructose and glucose (Fig. 6b), which is consistent with the lack of transport activity reported earlier suggesting that mannitol uptake is not inducible by fructose. The *smoK*, *frcA* and *smoK/frcA* mutants showed ^14^C-mannitol transport rates like wild-type (Fig. 6b). Interestingly, the *frk* and *frcK* strains showed ^14^C-mannitol transport rates well over background (Fig. 6b), *frk* rates are approximately equivalent to mannitol grown wild-type cells and *frcK* rates are approximately 50 % of wild-type. That these strains exhibit mannitol uptake in a non-induced (fructose grown) state indicates that they can overcome repression of mannitol transport genes.

## Discussion

**Fig. 7.**
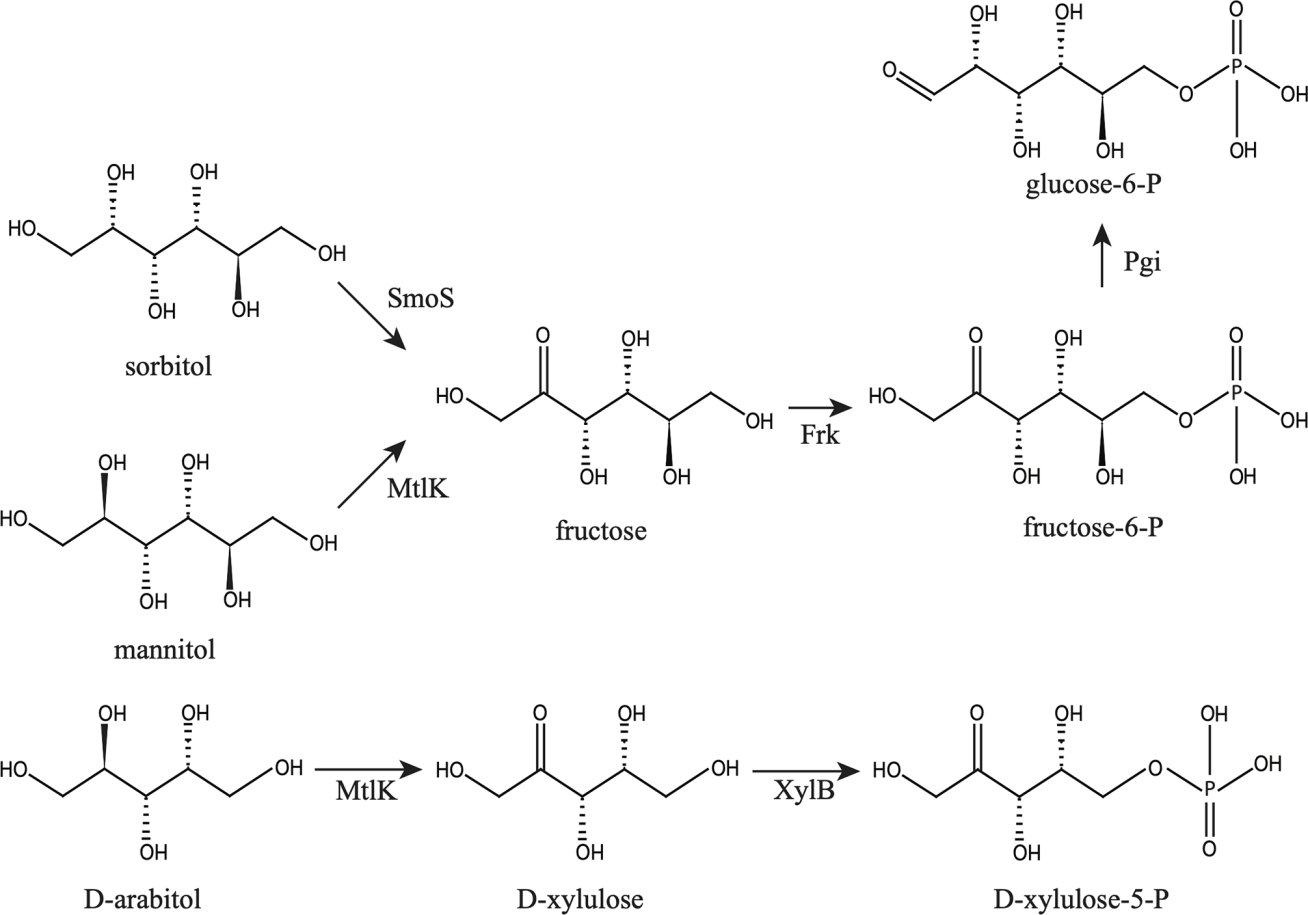
Pathways for the metabolism of sorbitol, mannitol and d-arabitol. Sorbitol and mannitol are oxidized to fructose by SmoS and MtlK, respectively. Fructose generated may be mutorotated by FrcS before phosphorylation into fructose-6-phosphate by Frk and isomerization by Pgi. d-arabitol becomes d-xylulose, catalysed by MtlK, which is subsequently phosphorylated by XylB into d-xylulose-5-phosphate.

In this work we show that *smoS* and *mtlK* encode determinants necessary for the oxidation of sorbitol and mannitol to fructose (Fig. 2). In addition, MtlK is shown to be able to use d-arabitol as a substrate yielding d-xylulose. Whereas the fructose that is generated by either SmoS or MtlK is dependent upon the gene products of *frk* and *pgi* to be integrated into central metabolism, d-xylulose is dependent on *xylB* [38] (Fig. 7). Additionally, fructokinase activity has been ascribed to Frk (Fig. 5), and experimental evidence has been provided for the role of *pgi* (Table 3).

It has been shown that the substrate-binding protein of the *smo* transporter, SmoE, is induced by sorbitol, mannitol, d-arabitol and maltitol [42]. Sorbitol and d-arabitol compete with mannitol for use of the *smo* transporter, and maltitol exhibits low-affinity transport in conjunction with mannitol (Fig. 3). That growth on maltitol is reduced in a *smoS* mutant background suggests that SmoS is involved in the catabolism of maltitol. We hypothesized that maltitol is being hydrolysed by an unknown glucosidase into glucose and sorbitol, with the glucose monomer able to support the growth of a *smoS* mutant. Attempts at identification of this glucosidase via Tn*5* mutagenesis have been so far unsuccessful. However, a blast search of the maltitol degrading α-glucosidase PalH sequence from *Erwinia rhapontici* [49] against the *S. meliloti* genome identified two genes, *agaL2* and *melA*, as likely to encode a similar protein. Additionally, *S. meliloti* trehalose transport, *thuEFGK*, and catabolic genes, *thuAB*, have been implicated in the utilization of maltitol via a non-specific catabolic route [50]. These genes make compelling targets for future work on maltitol metabolism in *S. meliltoi*.

While the catabolic pathways for sorbitol and mannitol are characterized and straight forward, the transport of these compounds is less clear. ABC-type transporters are typically characterized as being tripartite in nature containing a periplasmic solute binding protein, permease components, as well as an ATP binding protein. These transporters are generally regarded as high-affinity transporters with *K*_M_s in the micromolar range and consequently are used to transport a single solute [51]. Whereas this is often the case, it is noteworthy that several *S. meliloti* transporters have been reported to transport multiple substrates including erythritol, ribitol, and l-arabitol by MptABCDE [38], as well as transport of galactose and l-arabinose through AraABC [28].

The evidence for sorbitol and mannitol utilizing a second transporter for uptake is based on the fact that if a multicopy plasmid containing either *smoS* or *mtlK* is introduced into a strain carrying a *smo* transporter mutant, it is capable of restoring growth (Table 2), a strain carrying a *frcA* or *frcC* mutation exhibits slow growth on fructose, mannitol and sorbitol (Table 4), and finally strains carrying mutations in both the *smo* and *frc* transporters are completely unable to grow on sorbitol, mannitol or fructose despite the presence of metabolic genes on a plasmid (Table 4).

Collectively these data make a strong case that both transporters can transport all the listed substrates, however we do not believe that they transport all the substrates with equivalent efficiency. In all cases where a multicopy plasmid was introduced, it is very likely that the overall equilibrium of the pathway might be shifted to favour catabolism, as previously demonstrated with rhamnose catabolism in *R. leguminosarum* [47]. The fructose transporter has been shown to have an apparent *K*_M_ of 6 µM [17], which is consistent with it being a high-affinity transporter. Even though fructose can inhibit mannitol transport, it was far less competitive than sorbitol, mannitol or d-arabitol (Fig. 3b). Finally, a strain carrying a mutation in the *smo* transporter had a growth rate equivalent to that of the wild-type when grown on defined medium with fructose (Tables 2 and 4). Collectively these data suggest that although sorbitol and mannitol can utilize the *frc* transporter, and that fructose can utilize the *smo* transporter, it is more likely that the uptake systems behave as low-affinity transporters for these substrates, which is evident only when conditions are manipulated to allow this to happen. It is noteworthy that the substrate concentrations used in transport experiments is 2 µM whereas the plate concentration of substrates is 15 mM. This difference in substrate concentration on agar plates may facilitate the ability of these transporters working as low-affinity transporters.

The fructose locus of *S. meliloti* consists of genes annotated as *frcA*, *frcB*, *frcC*, *frcK*, *frcR* and *frcS* (Fig. 1b). The genes *frcRS*, which encode a putative negative regulator and a mutorotase, are divergently transcribed from *frcB*, and *frcCAK*. Two differentially promoters are recognized for *frcB* and *frcCAK*. The first, is upstream of *frcB* (which encodes the solute binding protein) and is expressed constitutively [42]. The second is upstream of *frcCAK*, which encodes a permease, an ABC protein and a kinase respectively, and is inducible by mannitol and fructose [17].

The transport of fructose by the *frc* locus has been characterized, however our data are not entirely consistent with previously reported results. Prior fructose transport mutations (*frcC*) were reported to abolish growth on fructose with no effect on mannitol utilization or transport [17]. Our data indicate that *frc* mutants exhibit reduced growth on sorbitol, mannitol and fructose, and that transport of these substrates is shared between the *smo* and *frc* transporters (Table 4). We note that the strains and method of mutagenesis differ between previous work and our own, though how these differences could account the phenotypic discrepancies is not clear.

Consistent with previous results, we determined that growth on fructose or mannitol induces the uptake of fructose [17]. Additionally, we showed that growth on fructose does not induce transport of mannitol, but that mannitol transport occurs following growth on fructose in either a *frcK* or *frk* mutant backgrounds (Fig. 6b). While Frk is definitively a fructose kinase (Fig. 5), the role of FrcK is less clear. The *frcK* gene is annotated as being a putative fructose transport system kinase. While FrcK shares little sequence similarity with known sugar kinases, a conserved domain search on NCBI yielded two specific hits, which are to the P-loop containing NTP hydrolase superfamily (E value 3.73×10^−105^) and to pantothenate kinase (E value 1.8×10^−85^). Mutations to *frk* or *frcK* appear to lift a regulatory hinderance to mannitol uptake imposed by growth on fructose. The ability of mannitol to be taken up in *frk* and *frcK* mutant backgrounds may occur due to the presence of a metabolic block, which alters internal metabolite pools, perhaps allowing the accumulation of fructose or a decrease in the F6P levels. The idea that these metabolites directly affect the regulation of the *smo* operon has not been subject to a thorough investigation.

Strains with mutations to *frcK* exhibit a reduced ability to transport fructose (Fig. 6b), suggesting that it has a role in fructose uptake. However, *frcK* mutants also exhibit reduced growth on sorbitol and mannitol, which can be restored by the introduction of *frk* on a plasmid (Table 4). Additionally, *frcK* mutants permit the transport of mannitol in a manner like *frk* mutants (Fig. 6b). Although the results suggest that these phenotypes are functionally linked, the specific role of *frcK* is unclear. It may be that FrcK affects the activity of the transporter(s) through a direct or indirect interaction. We note that kinases that affect transporter activity have been previously described in rhizobia [37, 52]. The substrate for the putative kinase activity of FrcK is not readily apparent from our work.

The relationship between sorbitol/mannitol transport and fructose transport was unexpected. Although there are few metabolic steps involved in converting either sorbitol or mannitol to fructose, the oxidation of either compound will initially yield a linear form of fructose, which will need to cyclize and possibly mutorotate prior to being phosphorylated by Frk. We note that *frcS* encodes a putative mutorotase. The interaction of these metabolites, and their stereochemistry, with the two potential regulators (SmoC/FrcR) suggests a more complex level of regulation that warrants further investigation.

## Supplementary Data

Supplementary material 1
