# Supplementary material for: The transport of mannitol in Sinorhizobium meliloti is carried out by a broad-substrate polyol transporter SmoEFGK and is affected by the ability to transport and metabolize fructose
Source: Microbiology (Reading). 2023 Jul 28;169(7):001371. doi: 10.1099/mic.0.001371 (PMC10433430; doi:10.1099/mic.0.001371)
Supplement: Supplementary material 1 [file mic-169-1371-s001.pdf]

Table S1. Primers used during this study.

Name	Sequence 5′→3′
Generation of mutants	
*frk*_pK_F	ATAT**GGATCC**AGCTCGTCGACGGGCATGC
*frk*_pK_R	ATAT**CTCGAG**CGTCCGCGCCGCGGGTG
*frcK*_pK_F	ATAT**TCTAGA**GCTTGCCCGCAAGGGGGC
*frcK*_pK_R	ATAT**GGATCC**CGCTTCGTCGAGCAGCAGG
*smoC*_pK_F	ATAT**TCTAGA**TCGACCACCGTGGGCATC
*smoC*_pK_R	ATAT**CCCGGG**TCTTCGCAAAGCGGCGCG
*pgi*_F1	ATAT**TCTAGA**TCGAGACGATGACCAATGCG
*pgi*_R1	ATAT**CCCGGG**GCCGGAAAGCGCGTCAGC
*frcA*_pK_F	ATAT**GGATCC**ACGGTCTATCAGAACCTCGC
*frcA*_pK_R	ATAT**CTCGAG**TGGATGGTCATCAGACCGAG
*frcC*_pK_F2	ATAT**GGATCC**CATCATGGGGCAGTTCACCT
*frcC*_pK_R2	ATAT**CTCGAG**CATAGACATAGCGCCCCCAG
Confirmation of mutants	
*frk*_F	ATGATCGTTTGCTGCGGAGAG
*frk*_R	TCGGAGCCCGAGTTCGTGTC
*frcK*_F	ATGAGCGTCAAATCCCTTGCG
*frcK*_R	CTAAAAGTCCCTGATCACGAC
*smoC*_F	ATGGCACGCAAGGCGGAAAG
*smoC*_R	CTAGACCCGGAGCAGATAGTC
*pgi*_F3	CTTCATCGTCGCCTCCAAGA
*pgi*_R3	ATGACATCGGTTCCCTGGTG
*frcA*_F2	GGCACAGGAACCCATTCTCA
*frcA*_R2	TGGGATTGATGACGCAGAGG
*frcC*_F2	AGCACTTCCTGCATTCGAGC
*frcC*_R2	AGCAAGCCGATCAGGAGATA
Generation of expression constructs	
*frk*_F2	ATATA**AAGCTT**GGAGATGCATGCATGCACCACCACCACCACCA CATCGTTTGCTGCGGAGAGGC
*frk*_R2	ATAT**GAATTC**TCAGAGCCCGAGTTCGTCTC

Sequence used for primers used in the study are presented as 5’ to 3’. Primers that were used to generate mutants or expression constructs have added restriction sites to facilitate cloning are bolded.
